# Pediatric Aspects of Nutrition Interventions for Disorders of Gut-Brain Interaction

**DOI:** 10.14309/ajg.0000000000001779

**Published:** 2022-04-13

**Authors:** Samuel Nurko, Marc A. Benninga, Toni Solari, Bruno P. Chumpitazi

**Affiliations:** 1 Center for Motility and Functional Gastrointestinal Disorders, Boston Children's Hospital, Boston, Massachusetts, USA;; 2 Department of Pediatric Gastroenterology, Emma Children's Hospital Amsterdam UMC, Amsterdam, the Netherlands;; 3 Department of Pediatrics, Baylor College of Medicine, Houston, Texas, USA;; 4 United States Department of Agriculture, Children's Nutrition Research Center, Houston, Texas, USA.

## Abstract

Dietary factors may play an important role in the generation of symptoms in children with disorders of gut-brain interaction (DGBIs). Although dietary modification may provide successful treatment, there is a relative paucity of controlled trials that have shown the effectiveness of dietary interventions. This study is a narrative review that explores the existing literature on food and pediatric DGBIs. The following have been shown to be beneficial: (i) in infants with colic, removing cow's milk from the infant's diet or from the maternal diet in those who are breastfed; (ii) in infants with regurgitation, adding thickeners to the formula or removing cow's milk protein from the infant's diet or the maternal diet in those who are breastfed; and (iii) in children with pain-predominant DGBIs, using soluble fiber supplementation or a low fermentable oligosaccharides, disaccharides, monosaccharides, and polyols diet. In children with functional constipation, there is no evidence that adding fiber is beneficial. Given that most dietary interventions include restriction of different foods in children, a thoughtful approach and close follow-up are needed.

## INTRODUCTION

Disorders of gut-brain interaction (DGBIs) are very common in children (1–5). Food may have an important role in the generation of symptoms, and therefore, dietary modification may provide successful treatment (1,6–9). The pathophysiology of food-induced problems in DGBIs in children is complex and multifactorial and includes several behavioral (psychological and social) and biological factors (physiological effects of diet, food intolerance, gut microbiome, visceral hypersensitivity, central and peripheral sensitization, and dysmotility) (1,10). This study is an expert narrative review that addresses the relevant information regarding the role of food and pediatric DGBIs.

## METHODS

This narrative review was performed after doing an extensive review of the literature on dietary interventions in pediatric DGBIs using PubMed (2,3). Controlled and uncontrolled trials, systematic reviews, meta-analyses, and review articles were identified. The authors discussed, reviewed, and summarized the identified references to achieve consensus on the provided recommendations. This review will focus only on specific dietary interventions (food) in those pediatric DGBIs in which dietary interventions have been studied in controlled trials.

## INFANT COLIC

Infant colic has been described as a behavioral syndrome of early infancy involving long periods of crying and hard-to-soothe behavior (7,10,11). It affects somewhere between 4% and 28% of infants worldwide and usually resolves by the age of 5 months (10).

The etiopathogenesis of infant colic remains undefined (10). Several factors can contribute to its manifestation including excessive gas production, dysbiosis, gut inflammation, alterations in motility, food intolerance or allergy, and enteric nervous system immaturity and behavioral factors (7,10,12,13).

### Dietary therapies for children with colic

**Figure 1.**
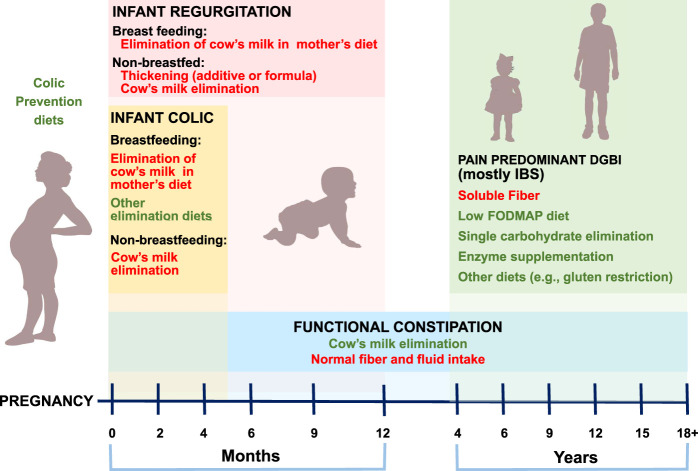
Specific dietary interventions for pediatric disorders of gut-brain interaction (DGBIs). Specific DGBIs are placed according to the age of presentation. Evidence-based dietary interventions are shown in red. Those with some information are shown in green. IBS, irritable bowel syndrome.

Most dietary interventions have focused on either changing the infant's formula in non-breastfed infants or changing the maternal diet on those babies that are exclusively breastfed (10) (Tables 1 and 2; Figure 1).

**Table 1.**
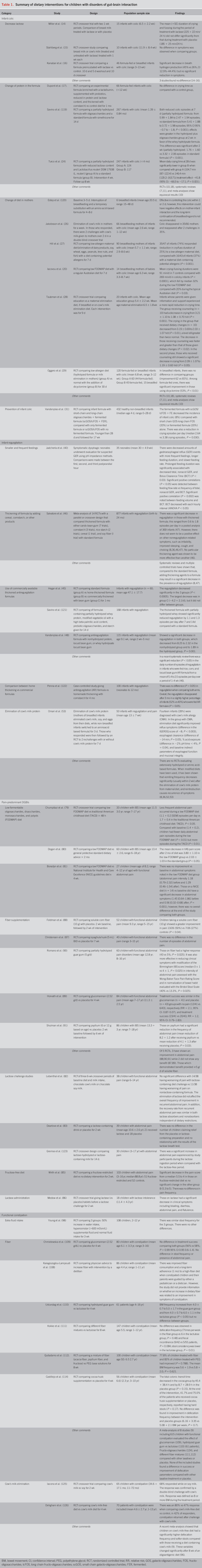
Summary of dietary interventions for children with disorders of gut-brain interaction

**Table 2.**
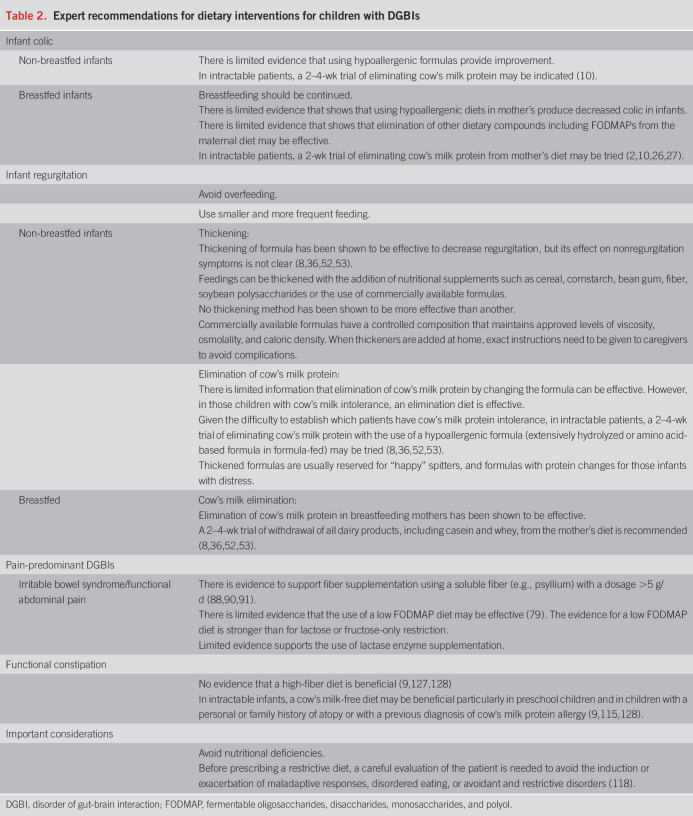
Expert recommendations for dietary interventions for children with DGBIs

#### Non-breastfed infants.

Modifications to the infant formula involve changing the carbohydrate (hypothesizing that malabsorption of lactose may lead to increased gas production, fussiness, and crying) or changing the protein content or type (hypothesizing that colic is secondary to intolerance or allergy) (10). Decreasing the lactose content has not been shown to produce a difference in symptom reduction (7,14–16). Attempts to alter the protein intake have included either modifying the amount of protein or modifying its type by using specialized formulas (soy-based, partially hydrolyzed, extensively hydrolyzed, amino acid-based/elemental, the addition of prebiotics, etc.) (7,10).

1. Modifying the amount of protein: Double-blind randomized controlled trial (RCT) studies have failed to show a difference in the amount of crying (7,17).
2. Soy-based formulas: They may reduce symptoms of colic (18). However, the European Society for Pediatric Gastroenterology, Hepatology, and Nutrition stated recently that there is insufficient evidence to support the use of soy formulas for colic. In addition, owing to concerns regarding a cross-over allergy to cows' milk protein and their estrogen content, it is recommended that soy-based formulas should not be given to infants (19).
3. Hypoallergenic formulas: Studies have shown conflicting results (7,20–24). Recent systematic reviews and meta-analyses that have included more than 15 RCTs and 1,200 infants have concluded that although there is insufficient evidence to recommend dietary modifications in all infants with colic, a 2–4-week elimination diet may be indicated in more severe cases, given the possibility of an underlying cow's milk allergy (CMA) (2,7,10,21,22).

#### Breastfed infants.

Breastfeeding should be continued. Mothers often alter their diet in an attempt to settle their infant, commonly by reducing intake of dairy and intestinal gas-producing foods, especially pulses/legumes, onion, garlic, cruciferous vegetables, wheat, and rye.

There is some experimental evidence on the association between maternal intake of cow's milk and crying in colicky infants (11,25), and several studies have demonstrated a reduction in colic when breastfeeding mothers consumed a hypoallergenic diet (26,27). A recent trial randomized breastfeeding mothers of babies with colic to either a low fermentable oligosaccharides disaccharides monosaccharides and polyols (FODMAP) diet or a regular Australian diet and showed a significant reduction in crying time for the low-FODMAP group (20).

It is important to note that other nondietary interventions such as education or medication may be as or more effective than dietary changes (28,29).

#### Prevention of infant colic.

There are no available RCTs evaluating whether maternal avoidance of cow's milk or other dietary interventions during pregnancy or after delivery will prevent colic. Studies have failed to show a protective effect of maternal diets during pregnancy on infant development of allergic problems, including CMA (30). A recent RCT demonstrated that a combination of fermented formula with galacto-oligosaccharides and fructo-oligosaccharides modestly decreased the incidence of infant colic (8%), but more information is needed before this can be recommended (31).

## INFANT REGURGITATION

Infant regurgitation is one of the most frequent DGBIs in children with a prevalence of about 20% (2,8,32,33). Gastroesophageal reflux (GER) is a normal physiologic event that occurs multiple times a day and manifests itself as infant regurgitation (8,34). GER can evolve into a pathologic entity, gastroesophageal reflux disease (GERD), when it becomes troublesome and symptomatic or is associated with esophageal damage or extraesophageal problems (8,35).

Factors that contribute to the more frequent physiologic reflux in an infant include a combination of large fluid intake, a shorter esophagus, a small stomach size and capacity resulting in faster gastric distention and increased intragastric pressure, frequent feedings per day, an exclusively liquid diet, high fluid intake per kilogram per day, and a supine position that predisposes to a common immersion of the gastroesophageal junction, compounded by a small esophageal capacity to hold fluids (2,8,36–42). The type of diet can also affect esophageal and gastric functions (37,38). Breastfed infants have less regurgitation in comparison with formula-fed children (38), and hypoallergenic formulas have been shown to decrease reflux events and enhance gastric emptying in comparison with regular infant formulas (8).

### Dietary therapies for infants with regurgitation

Treatment of infant regurgitation should be tailored to the child's clinical presentation, psychosocial circumstances, and underlying pathophysiological mechanisms (2,8) (Tables 1 and 2 and Figure 1). No medications are necessary, and multiple RCTs have shown that proton pump inhibitors are not effective in controlling the regurgitation or irritability, they increase adverse events and should not be given to children with infant regurgitation (2,8,43,44). The treatment of infant regurgitation includes mostly conservative measures.

#### Reduction of the ingested volume of formula.

Although there are no RCTs, frequent feedings of smaller volumes for age while maintaining an appropriate total daily amount of formula or breast milk to meet the child's nutritional needs are often recommended (8,36,37,45).

#### Thickening.

The next consideration, especially if there is inadequate weight gain, includes formula thickeners (8,37,46). Thickening decreases visible regurgitation and therefore provides increased calories, and it has an impact on achieving parental reassurance (46). The impact on nonregurgitation symptoms is less clear (8,36,37,47,48).

There are 2 main approaches to thickening: (i) home thickening of a standard formula (which is usually less expensive) or (ii) commercially available thickened formulas (46).

*Thickeners* that have been added to formulas include cereal, cornstarch, bean gum, fiber, soybean polysaccharides, and other commercial products (46). Care should be taken when adding thickeners because the osmolarity and viscosity of the formulas may change to nonrecommended levels (46). One study reported that a heaping tablespoon of starch added a quantity between 3.6 g and 4.6 g (49). This is well above the regulatory limit for starch in antiregurgitation formulas; it increases formula osmolarity and provides an additional 20 calories per 100 mL. Furthermore, overthickening results in a higher viscosity, requiring an increased sucking effort and/or a crosscut nipple to flow through (46). No one particular thickening agent is more effective than another.

There have been safety concerns regarding the high levels of inorganic arsenic in rice cereal, which may cause neurotoxicity and increase long-term cancer risk. In April 2016, the US Food and Drug Administration proposed a limit of 100 parts per billion for inorganic arsenic in infant rice cereal, which corresponds to a level proposed by the European Commission for rice destined for the production of food for young children. The relatively large volume of rice cereal needed to thicken infant formulas puts this patient population more at risk for high arsenic levels. Despite the warning, thickening with rice cereal does have some advantages over other cereals, including its high solubility, low cost, and successful long-term use. Prospective studies are underway to try to better clarify these issues. Cereal cannot be used to thicken breast milk because of the presence of amylases.

There are other commercial thickeners such as xanthan gum-based, carob-based, and cornstarch-based thickeners that can be used to thicken breast milk (50). Concerns have been raised about the risk of thickeners in infants, including arsenic exposure, necrotizing enterocolitis, dehydration, decreased intake, and constipation, and these concerns sometimes limit their use (50). Whenever a thickener is used, clinical follow-up is needed to ensure that patients tolerate the degree of thickening with adequate improvement in symptoms and minimal adverse effects.

*Commercially prepared thickened formulas* are at times preferred because they usually have better viscosity, digestibility, and nutritional balance (46). Commercial antiregurgitation (AR) formulas have a controlled composition with thickening components less than 2 g/100 mL for starch and 1 g/100 mL for carob bean gum and a caloric content that is similar to a standard formula (46). Locust bean gum increases viscosity more than other thickening agents, but there is no clinical evidence that it is superior to other additives (51).

There are 2 types of commercial formulas—AR “regular” formulas or “comfort” formulas. While the first is positioned to reduce regurgitation in the “happy spitter,” the second is positioned in the management of the infant presenting with regurgitation and distress. These comfort formulas contain partially hydrolyzed proteins and are reduced in lactose content (36). A thickened extensive hydrolysate may be effective, independent of the cause of the symptoms, but does not make a precise diagnosis of CMA, given other effects on esophageal and gastric physiology (36,52).

#### Elimination of cow's milk protein.

GER and CMA may both occur in the first year of life. Differentiating between troublesome GER symptoms, GERD, and CMA may be challenging because the symptoms overlap. The association of CMA with GERD has been reported in 16%–56% of cases with persistent gastrointestinal symptoms and suspicion of GERD (52). It has also been reported that CMA can induce GER (53). Elimination of cow's milk may significantly improve reflux symptoms, esophageal clearance, and baseline indirect parameters of esophageal function and mucosal integrity (53).

Elimination of cow's milk protein has been achieved either with the use of extensively hydrolyzed or amino acid-based formulas or with the elimination of cow's milk protein intake in mothers of breastfed infants (52). When the symptoms are due to allergy, extensively hydrolyzed formula reduces GER symptoms and vomiting frequency (usually within 2 weeks), and reintroduction causes recurrence of symptoms (8,36,52,53). In non-breastfed infants with suspected CMA, formulas with cow's milk-based extensively hydrolyzed proteins can be tried as the first choice, rice hydrolysates are the second option, and amino acid-based formulas should be reserved for more severe clinical reactions (52). There are no specific RCTs that evaluate extensively hydrolyzed or amino acid-based formulas for regurgitation. In breastfed infants, elimination can be achieved if the mother eliminates all dairy ingestion including casein and whey products (8,36,52).

Given the similarity of the symptoms between infants with severe regurgitation and CMA and the difficulty in making the diagnosis, a trial of a minimum of 2 weeks with an extensively hydrolyzed formula or amino acid-based formulas or avoidance of cow's milk by breastfeeding mothers may be indicated in infants who have not responded to conventional therapies.

## PAIN-PREDOMINANT DISORDERS OF GUT-BRAIN INTERACTION

The pediatric pain-predominant DGBIs affect approximately 13.5%–15.8% of children worldwide (3,54,55). There are several inter-related factors playing a role in an individual, including psychosocial distress, visceral hypersensitivity, gut microbiome, and diet (3,6,56–70). Diet is an important perceived inducer of gastrointestinal symptoms in children with DGBIs, and 92%–93% of those with irritable bowel syndrome identify at least 1 type of food trigger which exacerbates their symptoms (57,58) and a higher median number of foods causing gastrointestinal symptoms (58,59).

Children with food-induced symptoms report reduced daily intake of overall calories, fat, and lactose (57) and use several coping strategies including consuming smaller portions, modifying foods, not eating even when hungry, and avoiding offending foods (57–59). In children with irritable bowel syndrome, an increasing number of self-perceived food culprits are associated with more severe gastrointestinal symptoms such as more frequent abdominal pain episodes; increased pain intensity; decreased quality of life, including interference with school performance, sports, and social activities; and psychosocial abnormalities (somatization, anxiety, and functional disability) (58,59).

Several pathophysiologic mechanisms may play a role in symptom generation. For example, in children with functional dyspepsia, the feeling of bloating, fullness, and nausea correlates with the amount of gastric food retention (62,63). However, a strong relationship between gastric accommodation abnormalities and meal-induced gastrointestinal symptoms has not been identified (71).

The type of food, in particular carbohydrates, may also affect symptom generation (65). There are several factors associated with symptom generation with carbohydrates including (i) the amount ingested, (ii) ingestion with a meal, (iii) small intestinal enzymatic activity (e.g., disaccharidases), (iv) consuming the carbohydrate with microorganisms capable of metabolizing it, (v) the gut microbiome, and (vi) other host factors such as visceral hypersensitivity (6). Several large studies have identified biopsy-based disaccharidase deficiencies in children with DGBIs (66,67,72,73). However, currently missing in those with identified disaccharidase deficiencies are evaluations of specific postprandial gastrointestinal symptoms or responses to dietary therapies.

The gut microbiome composition of children also differs from that of adults and is dependent on dietary intake and other environmental factors (69,74). Growing evidence suggests that it plays a role within the paradigm of food-induced gastrointestinal symptoms (75). It has also been shown that subsequent changes in gut microbiome composition after a dietary challenge may also relate to food-induced symptoms (70).

### Dietary therapies for children with pain-predominant DGBI

Although not supported by RCT evidence, given a relationship of symptoms to gastric emptying, clinical practice recommendation for children with functional dyspepsia is often to administer a low-fat diet and frequent meals (3) (Tables 1 and 2 and Figure 1).

#### Low FODMAP diet.

FODMAP carbohydrates include fructose, lactose, fructo-oligosaccharides (e.g., inulin), galacto-oligosaccharides, and polyols (76). Uncontrolled studies have identified improvement using a low FODMAP diet in children with pain-predominant DGBIs ranging from 50% to 79% (77,78). Two RCTs have identified amelioration of gastrointestinal symptoms using a low FODMAP diet vs either traditional diet or general protective standard diet (79,80). A small RCT did not identify a difference in abdominal pain frequency comparing a low FODMAP diet vs a National Institute for Health and Care Excellence diet (which also restricts fermentable carbohydrates) (81).

#### Single carbohydrate restriction.

Lactose restriction is often used and is supported by several uncontrolled studies. However, most RCTs evaluating lactose challenges in children with pain-predominant DGBIs have been negative (82,83).

Several pediatric uncontrolled studies suggest that a fructose-restricted diet may be helpful (84). One prospective RCT compared a two-week fructose-restricted diet vs no dietary intervention (85). Those on the fructose-restricted diet had less severe pain without a decrease in pain frequency (85).

#### Enzyme supplementation.

Administering lactase tablets before a lactose challenge decreased bloating, diarrhea, abdominal pain, and flatulence (86).

#### Fiber supplementation.

Five fiber supplementation RCTs, each using different fibers and/or amounts, have been conducted (87–91). The 3 RCTs which demonstrated improvement in gastrointestinal symptoms provided ≥5 g per day of soluble dietary fiber.

#### Nonceliac gluten sensitivity.

A study to determine the prevalence of nonceliac gluten sensitivity in children with pain-predominant DGBIs found that of 1,114 children without celiac disease or wheat allergy, 96% did not have a correlation of symptoms with gluten ingestion (92). Of the remaining 36 children with potential gluten sensitivity, only 11 (comprising <1% of the entire cohort) ultimately met double-blind placebo-controlled criteria for gluten sensitivity (92). Controlled long-term gluten-free diet studies in children with DGBIs without celiac disease or wheat allergy remain to be completed. In addition, studies related to whether fructans rather than gluten induce symptoms in children with DGBIs with suspected nonceliac gluten sensitivity remain to be completed (92).

## FUNCTIONAL CONSTIPATION

Functional constipation is a common condition in all pediatric age groups with a worldwide prevalence that varies from 3.0% to 14.4% (1–5,55,93). Although a clear explanation for this variation is lacking, dietary factors, different toileting behaviors, and other cultural-dependent differences in parent-child interactions may be contributing.

Defecation frequency and stool consistency of young infants are influenced by their feeding mode. Breastfed infants pass more frequent and softer stools than formula-fed infants, and breastfeeding is considered to prevent constipation (94,95). During infancy feeding, changes such as the transition from breastfeeding to formula feeding or the introduction of solid foods often trigger the onset of functional constipation (95).

The pathophysiology of constipation is multifactorial. Common factors include diet, physical activity, psychological disorders, colonic sensorimotor disturbances, and pelvic floor dysfunction (2,94–96).

### Dietary therapies for children with functional constipation

Many healthcare professionals recommend dietary changes as a first step in the management of children with constipation (94) (Tables 1 and 2 and Figure 1).

#### Fluid intake.

Insufficient fluid intake or excessive fluid loss due to severe diarrhea, vomiting, or fever may lead to the hardening of stools (97). This applies particularly to infants, who are more susceptible to dehydration because of their small body weight and high turnover of fluids. Increasing fluid intake may soften stools. However, most colonic fluids are not ingested from the diet but are the result of intestinal secretion, and only a small portion of the fluids present in the colon are retained in the stools. One RCT assessing extra fluid intake in children with functional constipation showed insufficient evidence for a beneficial effect (98). Therefore, current pediatric guidelines for functional constipation do not recommend increasing fluid intake (94,99). An exception should be made for the extra fluid that is required for certain medications to be taken, such as polyethylene glycol. Indeed, a higher defecation frequency was reported in children treated with polyethylene glycol during a period of high fluid intake, as compared with a period with lower fluid intake (100).

#### Westernized diet.

The highest prevalence of childhood constipation is found in Western countries and the lowest in Asian countries (5). These data might suggest a potential role for the Western-type diet (i.e., high in saturated fat, sugar, dairy, and processed food and low in dietary fiber) (101). The Western diet is correlated with higher rates of overweight/obesity, although there are conflicting results on the association between excessive body weight and constipation in children (102). Therefore, the potential contributions of the Western diet on constipation need to be further defined.

#### Fat intake.

Olive oil is sometimes recommended to act as a lubricant and stool softener in infants and toddlers with constipation. However, triglycerides are almost completely absorbed in the small intestine and are therefore not likely to affect stool consistency or colonic transit. Therefore, except for children with malabsorption, olive oil does not reach the colon to be able to exert a laxative effect (103).

#### Fiber intake.

Fiber is an essential nutrient in the human diet that is crucial for human health (103–105). Several studies from all parts of the world have shown that children consume an insufficient amount of dietary fiber (94,105,106). However, RCTs and meta-analyses have failed to show a benefit to the addition of fiber compared with placebo or laxatives (9,94,105,107–114).

A number of new prebiotic-fiber combinations provide some promising results. Further well-designed high-quality RCTs are needed before additional fiber intake can be recommended (9). Adding certain fibers to the diet may increase abdominal pain and flatulence, but the symptoms often decrease after several days. Sometimes gaseousness can be reduced by switching to another fiber supplement (9).

#### Cow's milk avoidance.

Scientific evidence regarding a causal relationship between functional constipation and CMA is controversial. In those who may have food allergy-related constipation, studies show that there is an increase in both rectal mast cell density and spatial interactions between mast cells and nerve fibers that correlate with anorectal motor abnormalities (96).

A review of 10 studies reported that a diet free from cow's milk resulted in an improvement in functional constipation in 28%–78% of children, with the first 3 years of life being the most affected age group (115). Meta-analysis evaluating cow's milk-free diet showed a significant effect of the cow's milk-free diet on treatment success and a significant improvement in stool frequency and consistency. The effect is particularly seen in preschool children and in children with a personal or family history of atopy or with a previous diagnosis of cow's milk protein allergy. The current European and North American pediatric gastroenterology society functional constipation guidelines recommend that a 2- to 4-week trial of cow's milk avoidance should be reserved for children who do not respond to conventional treatment (94).

## ADDITIONAL PEDIATRIC CONSIDERATIONS

Although dietary interventions may be helpful, it should be noted that using them for the management of DGBIs can have inadvertent side effects. Given the importance of nutrition in child growth and development, care to provide sufficient calories and nutrients when making dietary changes is paramount. This applies not only to restrictive diets where specific nutrients are being eliminated (116) but also when nutritional supplementation is being added, such as when a formula is being thickened. Given the varying nutritional needs based on the age and sex of children, a registered dietitian with pediatric expertise is an integral member of the healthcare team.

Besides malnutrition, dietary advice needs to take into account the child's psychosocial context. Young children are essentially completely dependent on caregivers for their dietary intake. Parental control of dietary intake diminishes as the child increases in age and reaches adolescence. Interventions, particularly in adolescents, should consider taking into account the child's perceived needs.

It should be noted that abnormal eating (e.g., excessive caloric restriction) can lead to debilitating GI symptoms. Given the need to consider a normal adaptive response to avoid foods that are perceived to trigger DGBI symptoms, determining when eating behaviors become disordered in a child, in particular an adolescent, can be challenging (117). Therefore, a careful assessment of the child and family's situation and dietary intake needs should be performed before dietary interventions are recommended. The recommendations to start a restrictive diet may be inappropriate when excessive food restriction is already taking place (117,118).

Recent information also suggests that there is overlap between patients with avoidance restrictive feeding disorder and DGBI both in adults and adolescents (118). In a recent study of pediatric patients with DGBIs aged 6–18 years, avoidance restrictive feeding disorder symptoms were present in 23%, and most frequently motivated by fear of aversive consequences, similar to findings in adults (118). Currently, there are no simple methods to differentiate between both, and the approach to treatment is different (117,118).

## CONFLICTS OF INTEREST

**Guarantor of the article:** Samuel Nurko, MD, MPH.

**Specific author contributions:** S.N., M.A.B., T.S., and B.P.C.: planning of the article, drafting the article, and reviewing and approving the final draft of the article.

**Financial support:** None to report.

**Potential competing interests:** M.A.B.: Consultant for Danone, FrieslandCampina, Sensus, United Pharmaceuticals and HIPP; B.P.C.: Receives royalties from the Rome Foundation for the use of the modified Bristol Stool Scale for Children; T.S.: No disclosures; S.N.: Consultant for Allergan and IHS.
